# Supplementary material for: Pilot Study of the Applicability, Usability, and Accuracy of the Nutricate© Online Application, a New Dietary Intake Assessment Tool for Managing Infant Cow’s Milk Allergy
Source: Nutrients. 2023 Feb 20;15(4):1045. doi: 10.3390/nu15041045 (PMC9960076; doi:10.3390/nu15041045)
Supplement: Supplementary file 1 [file nutrients-15-01045-s001.zip › nutrients-2138453-supplementary.pdf]

Supplemental Table S1 : Ratio of each food categories used to merge into one food category and their correspondence in macro-micro nutrient according to food composition database.

a) Animal-protein rich food

Food Categories	Ratio of intake *(%)*	Energy *(kcal/100g)*	Protein *(g/100g)*	Calcium *(mg/100g)*	Iron *(µg/100g)*
White meat	17.07	195	26	11.8	710
Red meat	33.37	173	28.1	9.43	210
Fish	17.37	106	22.1	27.6	90
Jam	23.02	117	20.5	14	380
Egg	9.17	134	13.5	41	60
**Mean Used***		**149**	**24**	**17**	**300**

*Mean used for Nutricate food composition database from Volatier el al [29]

b) Starchy

Food Categories	Ratio of intake (%)	Energy *(kcal/100g)*	Protein *(g/100g)*	Calcium *(mg/100g)*	Iron µg/100g)
Pasta	61.4	126	4.38	17	430
white rice	19.3	145	3.06	14	40
wheat semolina	19.3	122	3.75	13	1000
**Mean Used***		**128.9**	**4**	**15.7**	**460**

*Mean used for Nutricate food composition database from Volatier el al [29]

c) Vegetables

Food Categories	Ratio of intake (%)	Energy *(kcal/100g)*	Protein *(g/100g)*	Calcium *(mg/100g)*	Iron µg/100g)
Vegetable(cooked)	50	43.5	2.11	30.3	460
Tomato(not cooked)	50	19.3	0.86	8.14	120
**Mean Used***		**31.4**	**1.49**	**19.2**	**290**

*Mean used for Nutricate food composition database from Volatier el al [29]

d) Fruits

Food Categories	Ratio of intake *(%)*	Energy *(kcal/100g)*	Protein *(g/100g)*	Calcium *(mg/100g)*	Iron µg/100g)
Fruit raw	86.8	59.5	0.7	14.5	180
Fruit coocked	6.6	58.9	0.5	6.2	50
Fruit compote	6.6	102	0.5	18	130
**Mean Used***		**62.3**	**0.67**	**14.2**	**170**

*Mean used for Nutricate food composition database from Volatier el al [29]

Supplemental Table S2 : Macronutriments repartition intake (n=30)

Categories	Mean ± SD (%)
Proteins	13.5 ± 4.3
Lipids	28.2 ± 9.5
Carbohydrates	58.3 ± 10.7
